# Histidine Alleviates Impairments Induced by Chronic Cerebral Hypoperfusion in Mice

**DOI:** 10.3389/fphys.2018.00662

**Published:** 2018-06-07

**Authors:** Jiangman Song, Lu Yang, Di Nan, Qihua He, You Wan, Huailian Guo

**Affiliations:** ^1^Department of Neurology, People’s Hospital, Peking University, Beijing, China; ^2^Center of Medical and Health Analysis, Peking University, Beijing, China; ^3^Key Laboratory for Neuroscience, Ministry of Education, National Health and Family Planning Commission, Peking University, Beijing, China

**Keywords:** histidine, cerebral hypoperfusion, cognitive function, astrocyte, blood–brain barrier, cranial window

## Abstract

Chronic cerebral hypoperfusion is one of the fundamental pathological causes of brain disease such as vascular dementia. Exploration of effective treatments for this is of great interest. Histidine has been reported to be effective in anti-apoptosis, antioxidant, and against excitotoxicity. In the present study, we aim to investigate whether histidine could have a therapeutic effect on the impairments induced by chronic cerebral hypoperfusion. Cerebral hypoperfusion model was established through bilateral common carotid arteries stenosis (BCAS) operation in Tie2-GFP mice. Radial arm maze and Morris water maze revealed that histidine showed potential improvement of the tendency of cognitive impairments induced by hypoperfusion. The possible mechanisms were further investigated. After administration of histidine in hypoperfusion mice, immunofluorescent BrdU staining revealed more new-born nerve cells. *In vivo* observation through a cranial window under two-photon laser-scanning microscopy demonstrated that the blood flow velocity in capillary was improved, the distance between the astrocytes and the penetrating artery was shortened. Histidine administration also significantly increased the protein expression level of zonula occludens protein 1, an indicator of the integrity of blood–brain barrier (BBB). These results suggest that histidine could alleviate the impairments induced by chronic cerebral hypoperfusion in mice, and this effect may be related to the neurogenesis, astrocytes, and the integrity of the BBB.

## Introduction

Chronic cerebral hypoperfusion is a common consequence of arthrosclerosis (Choi et al., 2007). It is also the second primary cause of dementia, which is called vascular dementia. The Alzheimer’s disease, Binswanger disease and other neurodegeneration disease could be worsened under the condition of cerebral hypoperfusion (Jing et al., 2015; O’Brien and Thomas, 2015). Therefore, it is of great values to treat patients with chronic hypoperfusion. However, unlike the apparent symptoms in acute stroke, symptoms in the early stage of chronic hypoperfusion are mild and unnoticeable, but ingravescent (Ni et al., 1994). Nowadays, researchers realize the serious consequence of chronic and progressive hypoperfusion, and have interest in investigating therapeutics for the chronic cerebral hypoperfusion (Choi et al., 2016).

Histidine, a precursor of histamine, is a vital inflammatory agent in immune responses (Hu and Chen, 2017). It displays properties in anti-apoptosis, antioxidant and against excitotoxicity (Fang et al., 2014). Previous studies showed that it could prevent development of brain infarction (Adachi et al., 2005), and also improve myocardial dysfunction after ischemia/reperfusion injury (Cai et al., 1995; Alves et al., 2011). Histidine/histamine has various neurobiological functions, including modulating cognitive dysfunction in mice (Paul et al., 2002; Nozawa et al., 2014). A recent study demonstrated that histidine treatment showed the remarkable long-term neuroprotection with the reduction of the glial scar area and the astrocyte migration toward the infarct core (Liao et al., 2015). Based on the reports above, we hypothesize that histidine may exert a therapeutic effects for the impairments induced by chronic cerebral hypoperfusion.

In the present study, chronic cerebral hypoperfusion model was established through bilateral common carotid arteries stenosis (BCAS) operation. Effects of histidine on the cognitive functions were observed, and changes of blood flow velocity, new-born nerve cells, astrocytes and blood–brain barrier (BBB) integrity were analyzed to try to figure out the possible mechanisms underlying the histidine improvement.

## Materials and Methods

### Animals

Male Tie2-GFP mice [STOCK Tg (TIE2GFP) 287Sato/J] of 8–10 weeks (body weight 25–28 g) were used. The mouse is transgenic with the endogenous green fluorescent protein (GFP) expression in endothelial cells as previously described in our lab (Guo et al., 2011). All experiments were approved by the Animal Experimentation Committee of Peking University People’s Hospital and conducted in compliance with the National Institutes of Health Guide for the Care and Use of Laboratory Animals.

### Establishment of Chronic Cerebral Hypoperfusion Model in Mice

Mice were anesthetized with sodium pentobarbital (60 mg/kg) by intraperitoneal injection, and subjected to BCAS using microcoils (Sawane Spring, Japan) with inner diameter of 0.18 mm. Through a midline cervical incision, bilateral common carotid arteries (CCA) were exposed. One microcoil was twined by rotating it around the right CCA. After 30 min, another microcoil of the same size was twined around the left CCA (Shibata et al., 2004; Maki et al., 2011; Hattori et al., 2016). Sham-operated mice underwent the same surgical procedure without artery stenosis. Body temperature was maintained by a small-animal warmer/thermometer system (BWT-100A, Bio Research Center, Japan) during surgery.

### Histidine Administration

Mice were randomly assigned to three groups: the Sham-operation group (*n* = 27), Hypoperfusion group (*n* = 26), and Histidine group (*n* = 18). The mice in the Histidine group were underwent BCAS operation and treated with histidine. Histidine (1 mg/g body weight, Sigma, United States) dissolved in normal saline was given to mice after BCAS by intraperitoneal injection every other day until sacrificed. The dose of histidine was chosen on the basis of previous research (Liao et al., 2015). For the vehicle control of histidine, normal saline of the same volume was given to the sham-operated mice and the rest BCAS-operated mice. In the whole experimental period of 6 weeks, mice were housed in standard cages under a light cycle of 12 h with standard dry food pellets and water available *ad libitum*, unless otherwise noted.

### Radial Arm Maze Test

The radial arm maze was tested 6 weeks after the operation to assess the spatial working memory. The maze was consisted of eight arms (8 cm × 35 cm) that radiated from central starting point. One week before training, mice were given restricted diet until the body weigh decreased to 80% of the original level, so that they had enough motivity to search food in maze. Mice were allowed to explore the maze for 10 min at the first 3 days of pretraining. Subsequently, they were trained to find the food reward (cheese with a diameter of 1 mm) from the central starting point to each single open arm, in order to make the mice realize that each arm exists a piece of cheese. A trial was finished when mice were trained to find the food in eight different arms. Every mouse was trained two trials per day for 4 days. After these pretraining trials, mice were placed in the center starting point and allowed to search all the food in 8 arms for testing. One trial was finished when eight piece of cheese were all found or the time spent on searching was over 25 min. The test stage lasted 7 days, including 14 trials. The right choice was defined as the mouse came into a new arm and found the cheese correctly in the first eight choices. The wrong choice was defined as the mouse chose the arm it has already visited and there is no cheese left until the trial finished. The frequencies of correct and wrong entrances were both noted (Coltman et al., 2011).

### Morris Water Maze Test

Spatial reference learning and memory ability was evaluated by the Morris water maze 6 weeks after the hypoperfusion operation. Mice were placed in a large circular pool with a diameter of 150 cm. The pool was filled with water of 23°C at a depth of 30 cm. In initial trials, a visible platform was placed in the pool so that each mouse could reach from water. Visual cues were placed around pool so the mice could relate the spatial environment with the location of the platform. In subsequent trials, the platform was hidden 1 cm below the surface of water, and the escape latency that the mouse spent on finding the platform was recorded. The mice were trained four trials each day for acquiring the location of platform. To test memory ability, the amount of time and path length each mouse spent in searching in the target quadrant and the frequency each mouse crossed the target area were recorded in the last day of training with the platform removed.

### *In Vivo* Two-Photon Microscopy Observation of Blood Flow Velocity Through Cranial Window

Two-photon microscopy analysis was performed 6 weeks after operation. Cranial windows were implanted to facilitate the observation of brain tissue *in vivo* (Guo et al., 2011). Circular skull on the left parietal lobe was removed. A sterile glass cover slip was placed above the dura mater and sealed with dental cement (DMG, Hamburg, Germany). Two-photon laser scanning microscope (SP5, Leica, Germany) was used to image the blood stream. To obtain velocity of blood flow, line scan mode was chosen at 400 Hz (Huang et al., 2014), and 3–6 capillaries with a diameter less than 8 μm were selected every 50 μm depth from the brain surface to 400 μm beneath. The mean velocity of blood flow in capillary at each depth was calculated.

### *In Vivo* Two-Photon Microscopy Observation of Penetrating Arteries and Astrocytes Through Cranial Window

Astrocytes can be labeled by red fluorescence after injection of SR101 (8 μl/g, i.p.). Two hours after the injection of SR101, astrocyte somas became visible. The depth between 100–200 μm beneath the brain surface, equal to layer II/III of neocortex, was chosen to count the number of astrocytes because of the uniform distribution of astrocytes. The density of astrocytes, number of astrocytes in one stereoscopic cube (an arbitrary unit), was calculates 6 weeks after operation. The endogenous GFP expressed in endothelial cells can be used to locate blood vessels of the brain (Song et al., 2017). Penetrating artery is perpendicular to brain surface, and its location is relatively fixed (Sekiguchi et al., 2014). The distance between the penetrating artery and the nearest astrocyte soma was measured using 3D technique.

### Immunofluorescence Staining

For immunofluorescence staining, mice were sacrificed 6 weeks after operation. The brain was removed and fixed in 4% paraformaldehyde at 4°C for 24 h, then dehydrated with 20 and 30% sucrose. Frozen sections with a thickness of 30 μm were cut by cryostat (Leica 1900, Germany). The sections of all the groups were incubated with 5% BSA in PBS containing 0.3% Triton X-100 for 1 h at room temperature, then appropriate primary antibodies overnight at 4°C, rabbit anti-NeuN (1:500, CST, United States); rabbit anti-GFAP (1:1,000, Abcam, United Kingdom); goat anti-Iba1 (1:1,000, Abcam, United Kingdom); mouse anti-BrdU (1: 200, AbD, United Kingdom). After being washed in PBS, the sections were incubated with appropriate secondary antibodies for 1.5 h at room temperature. For labeling total cells, DAPI (1:5,000, Sigma, United Kingdom) was added to incubation buffer. The sections were observed with confocal microscope (Olympus FV1000, Japan).

### Western Bolt Assay

The integrity of the BBB was gauged through the expression level of zonula occludens protein 1 (ZO-1) by Western blot (Abbott et al., 2010). The tissue of parietal cortex was dissected and homogenized 6 weeks after operation. Western bolt was executed according to standard protocol with proper antibodies (Zhu et al., 2005). The hydrophilic polyvinylidene fluoride (PVDF) membrane was incubated in mouse anti-β-actin (1:1,000); rabbit anti-ZO-1 (1:500, Invitrogen, United States) overnight at 4°C. After washing with TBST buffer, the PVDF membrane was incubated with peroxidase-conjugated anti-rabbit antibody (1:1,000); peroxidase-conjugated anti-mouse antibody (1:1,000) for 2 h at room temperature. Relative densities of the bands were analyzed with software Quantity One 4.6.

### Statistical Analysis

Data were presented as Mean ± Standard Error of the Mean (SEM). Statistical analyses were conducted with the software GraphPad Prism 5.0. Data were analyzed with ANOVA followed by Turkey’s *post hoc* test for multiple measures. The escape latency of the Morris water maze test was analyzed by two-way ANOVA followed by Bonferroni *post hoc* test. A *p*-value less than 0.05 was considered to be statistically significant in all statistical analysis.

## Results

### Histidine Effects on the Tendency of Cognitive Impairments Induced by Hypoperfusion

**FIGURE 1:**
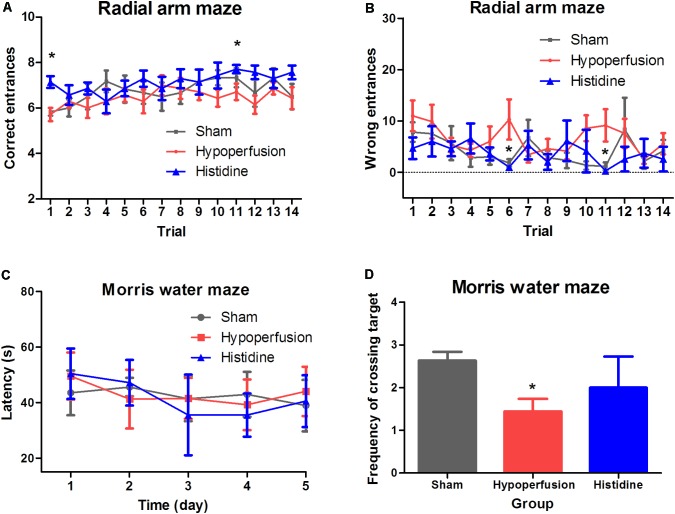
Effects of histidine on the cognitive dysfunctions induced by hypoperfusion. **(A)** The frequency of correct entrance was increased significantly after histidine administration (*n* = 6, ^∗^*p* < 0.05 compared with Hypoperfusion group). **(B)** The frequency of wrong entrance was decreased significantly after histidine administration (*n* = 6, ^∗^*p* < 0.05 compared with Hypoperfusion group). **(C)** The escape latencies of reaching the platform in Morris water maze showed no significant changes among the three groups (*n* = 6). **(D)** The frequency of crossing target was decreased significantly in Hypoperfusion group (*n* = 6, ^∗^*p* < 0.05 compared with Sham operation group). Histidine promoted the recovery of the decreased frequency after hypoperfusion slightly without significant difference.

Spatial working memory was assessed using the radial arm maze test. In the trial 1 and trial 11, a higher frequency of correct choice was shown in Histidine group, compared with that in Hypoperfusion group (*p* < 0.05, *n* = 6, **Figure 1A**). The more the mouse made the correct choice, the better the working memory function of the mouse was and vice versa. In the trial 6 and the trial 11, the mice in Histidine group showed lower frequency of wrong choice than that in Hypoperfusion group (*p* < 0.05, *n* = 6, **Figure 1B**). The less correct choices and more wrong choices in Hypoperfusion group than that in Sham operation group revealed dysfunction of spatial working memory after 6 weeks of hypoperfusion. These results suggest that histidine administration has a potential to alleviate the deficits of space memory induced by hypoperfusion.

The spatial reference learning and memory was examined using the Morris water maze test (**Figures 1C,D**). The escape latency to reach the target was measured. There were no significant differences among the three groups in the escape latency (*p* > 0.05, *n* = 6). The time and path length in quadrants were consistent with the escape latency, and the changes were not significant (data not shown). However, the frequency of crossing the target area in Hypoperfusion group (1.4 ± 0.3) was significant decreased compared with that in Sham operation group (2.6 ± 0.2) (*p* < 0.05). The frequency in the Histidine group was 2.0 ± 0.7, which was slightly higher than that in the Hypoperfusion group. But the difference was not statistically significant (*p* > 0.05). These results suggest that hypoperfusion for 6 weeks could lead a tendency of impairment in spatial reference learning and memory. Meanwhile, histidine could slightly, but not significantly alleviate this impairment.

### Histidine Increases Velocity of Blood Flow After Chronic Cerebral Hypoperfusion

**FIGURE 2:**
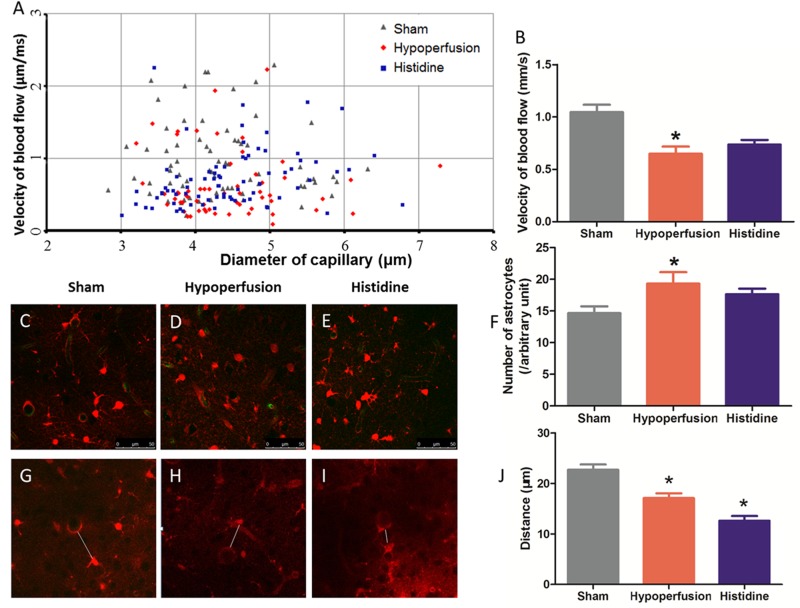
Effects of histidine on the blood flow velocity and astrocyte activation after hypoperfusion. **(A)** The blood flow velocity in different cortical capillaries was shown in three groups. One dot represents a value of velocity with a certain diameter. **(B)** The blood flow velocity was decreased significant after hypoperfusion (*n* = 9, 6, 6 respectively, ^∗^*p* < 0.05 compared with Sham operation group). Histidine slightly increased the blood flow velocity in Histidine group, but the difference was not significant. **(C–E)** Astrocytes were labeled with red color by SR101, and the number of astrocytes increased obviously after hypoperfusion. **(F)** The difference of astrocyte density between Sham operation group and Hypoperfusion group was significant (*n* = 9, 6, 6 respectively ^∗^*p* < 0.05 compared with Sham operation group). There was no statistical difference between Hypoperfusion group and Histidine group. **(G–I)** The distance between penetrating artery and the nearest astrocyte, which was represented by white line segment, was measured with 3D technology in three groups. **(J)** The distance decreased significantly after hypoperfusion (*n* = 6, ^∗^*p* < 0.05 Hypoperfusion vs. Sham). After histidine administration, the distance decreased further (*n* = 6, ^∗^*p* < 0.05 Histidine vs. Hypoperfusion).

The velocity of blood flow in cortical capillary was measured 6 weeks after hypoperfusion (**Figures 2A,B**). The velocity of blood flow of a capillary with a certain diameter was shown as a dot in **Figure 2A**. Blood flow velocities in capillaries were measured, so that the tendency in three groups became separable. The velocity in Hypoperfusion group (0.65 ± 0.07 μm/ms) was significantly lower than that in Sham operation group (1.05 ± 0.07 μm/ms) (*p* < 0.05, *n* = 6). This indicates that the mice in Hypoperfusion group underwent chronic cerebral hypoperfusion. The velocity of blood flow in Histidine group (0.73 ± 0.04 μm/ms) had a tendency of increase, compared with that in Hypoperfusion group (*p* > 0.05, *n* = 6).

### Histidine Induces Changes of Astrocyte After Chronic Cerebral Hypoperfusion

The changes of astrocyte were observed by two-photon laser-scanning microscopy *in vivo*. The astrocytes were labeled with red fluorescence by SR101, so the density could be calculated using 3D technology (**Figures 2C–F**). The density of astrocytes in the mice of Hypoperfusion group (2.9 ± 0.3 × 10^4^ cells/mm^3^, *n* = 6) was significantly higher (*p* < 0.05) than that in Sham operation group (2.2 ± 0.2 × 10^4^ cells/mm^3^, *n* = 9). The density of astrocytes in Histidine group (2.6 ± 0.1 × 10^4^ cells/mm^3^) was slightly less than that in Hypoperfusion group (*p* > 0.05, *n* = 6).

The change of the distance between the astrocytes and penetrating arteries was investigated (**Figures 2G–J**) by 3D reconstruction technique since long axis of penetrating arteries is perpendicular to brain surface. The distance in Hypoperfusion group was 17.12 ± 1.17 μm (*n* = 6), while the distance in Sham operation group was 23.90 ± 1.49 μm (*n* = 9). The distance between penetrating artery and the nearest astrocyte soma was decreased after hypoperfusion (*p* < 0.05). After the mice received histidine injection, the distance became 12.90 ± 1.12 μm, which was decreased further (*p* < 0.05, *n* = 6).

**FIGURE 3:**
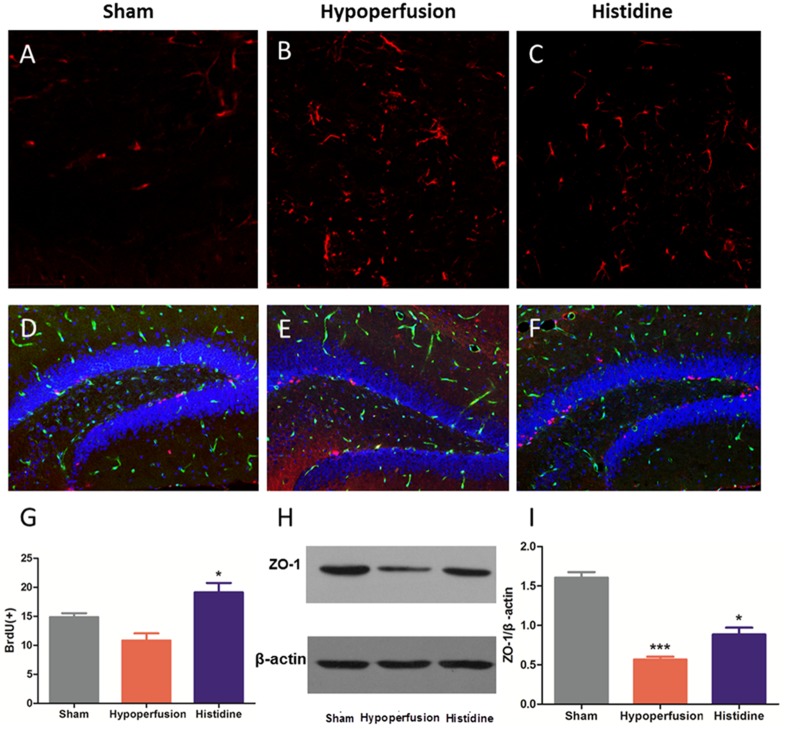
Histidine increases neurogenesis and ZO-1 protein expression. **(A–C)** Astrocytes (labeled with red fluorescence) were activated after hypoperfusion, and histidine decreased the activation. **(D–F)** New-born cells labeled with red fluorescence slightly decreased after hypoperfusion, which was reversed significantly by histidine (*n* = 6, ^∗^*p* < 0.05 Histidine vs. Hypoperfusion). Vascular endothelial cells were labeled with green fluorescent. All the cell nucleuses were labeled with blue fluorescence. **(G)** The new-born cells labeled with BrdU^+^ were increased significantly after histidine administration (*n* = 6, ^∗^*p* < 0.05 vs. Hypoperfusion). **(H)** The expression of ZO-1 was decreased after hypoperfusion, and partially recovered after histidine administration. **(I)** The expression level of ZO-1/β-actin decreased significantly after hypoperfusion (*n* = 6, ^∗∗∗^*p* < 0.05 Hypoperfusion vs. Sham). The expression level was increased significantly when using histidine (*n* = 6, ^∗^*p* < 0.05 Histidine vs. Hypoperfusion).

Using immunofluorescence staining, the astrocytes can be visualized in brain sections. In the staining of GFAP, the densities of astrocytes increased obviously after hypoperfusion. The dendrites of astrocytes in mice in Hypoperfusion group were thicker and longer than that in Sham operation group. In the Histidine group, the changes above were reversed (**Figures 3A–C**).

### Histidine Promotes Neurogenesis in Dentate Gyrus After Chronic Cerebral Hypoperfusion

The new-born nerve cells could be visualized in frozen brain sections by immunofluorescence staining for BrdU. The number of BrdU-positive cells in Histidine group (19.1 ± 1.6) was significantly increased compared with that in Hypoperfusion group (10.8 ± 1.2) (**Figures 3D–G**, *p* < 0.05, *n* = 6).

### Histidine Contributes to Maintaining the Integrity of the BBB

It was reported that decreased ZO-1 expression was consistent with the BBB disruption (Zhu et al., 2005). Protein expression of ZO-1 was significantly decreased after hypoperfusion for 6 weeks (*p* < 0.05, *n* = 6), whereas administration of histidine increased the ZO-1 expression (**Figures 3H,I** and **Supplementary Figures S1, S2**). These results suggest that the administration of histidine attenuated the BBB disruption induced by hypoperfusion.

## Discussion

In the present study, mice underwent BCAS operation to restrict the cerebral blood flow. The velocity of blood flow in cortical capillary was measured using two-photon microscopy *in vivo*. These results showed that the velocity of blood flow in the cortical capillary was decreased after BCAS operation (**Figure 2B**). This means chronic cerebral hypoperfusion model was successfully established by the BCAS operation.

Our model is just a hypoperfusion, not an ischemic infarction model. Open field test was performed to assess the motor function of mice with BCAS. The total distance between the Sham operation group and Hypoperfusion group was similar (data not shown), which proved motor function relatively intact after hypoperfusion for 6 weeks. Therefore, unlike the acute focal ischemia stroke, the deficits induced by BCAS limit to chronic cerebral hypoperfusion rather than ischemic infarction. Besides, TTC staining, H&E staining, and Nissl staining also confirmed that no focal infarction or obvious structural damage existed after hypoperfusion (data not shown). Thus, the chronic cerebral hypoperfusion in the present study is a type of injury with mild ischemia.

The cognitive function was examined through radial arm maze test and Morris water maze test. In radial maze test, the difference between the Sham operation group and Hypoperfusion group was significant in two trials out of 14 in the analysis of the correct entrances, as in that of the wrong entrances. In water maze test, the frequency of crossing the target area was different between the Sham group and Hypoperfusion group, while other parameters were not changed significantly, such as escape latency (**Figure 1**). Therefore, chronic cerebral hypoperfusion may lead a tendency of cognitive impairments. Other study also found this chronic and progressive hypoperfusion injury gradually causes cognitive function decline (Kitagawa et al., 2009). Nowadays, more and more researchers focus on the hypoperfusion injury.

On the basis of successful establishment of hypoperfusion model of mice we observed the effects of histidine. Histidine may ameliorate the tendency of cognitive impairments slightly. Histidine, a precursor of histamine, is an essential neurotransmitter. Previous studies suggested that histidine participated in regulation of nervous system inflammation (Frick et al., 2016) and thus provided long-term neuroprotection (Liao et al., 2015). In this study, the tendency of cognitive dysfunction induced by hypoperfusion seems to be alleviated after histidine administration (**Figure 1**).

We further investigate the possible mechanisms underlying the neuroprotection of histidine on the impairments induced by chronic cerebral hypoperfusion.

Firstly, histidine promotes neurogenesis in hypoperfusion mice. The BrdU staining showed that the new-born nerve cells in dentate gyrus increased (**Figures 3D–G**). The volume of the hippocampus decreased 7 months after chronic hypoperfusion with unilateral common carotid artery occlusion surgery (Zuloaga et al., 2015), which could account for the decline trend of cognitive function after chronic cerebral hypoperfusion. It has been reported that new-born neurons generated in adult brain on the granule layer of the dentate gyrus play a pivotal role in improving memory and learning (Lopez-Virgen et al., 2015). When histidine was administrated, the amelioration of the tendency of cognitive dysfunction may due to the neurogenesis in dentate gyrus.

Secondly, histidine shortens the distance between the astrocytes and the penetrating artery. Other study reported that histidine played a protective role after cerebral ischemia through promoting astrocyte migration *in vitro* (Liao et al., 2015). In the present study, the relative position between astrocyte and penetrating artery was measured. The distance between them was decreased after hypoperfusion, and this distance was decreased further after histidine administration (**Figures 2G–J**). Astrocytes, as a critical structural part of tripartite synapse and functional part of neurovascular unit, communicate with neurons and vessel endothelial cells (Liu and Chopp, 2016; Zhou and Liu, 2017). Thus, the relative position between the astrocyte and the penetrating artery may participate in the effects of histidine.

Meanwhile, according to our results, astrocyte proliferation is not involved in the histidine improvement. The astrocyte activation was detected by GFAP staining, and it was obvious after hypoperfusion (**Figures 3A–C**). When we calculated the density of astrocyte *in vivo* with two-photon microscopy, the density was significantly increased. That suggested that the hypoperfusion injury evoke the astrocyte proliferation. However, there was no significant change in the density of astrocyte between the Histidine group and Hypoperfusion group (**Figures 2C–F**, *p* > 0.05).

Thirdly, histidine increases the integrity of the structure of BBB. In consideration of neurovascular unit, astrocytes have a close relationship with vasculature and are also involved in construction of the BBB (Lee et al., 2003). In the present study, we found the histidine alleviated the destruction of the BBB induced by hypoperfusion (**Figures 3H,I**). Disruption of the blood–brain barrier has been proposed to be important in vascular cognitive impairment (Taheri et al., 2011), because it has been reported that the integrative BBB provides a stable environment for neural stem cells (Horgusluoglu et al., 2017). Thus, histidine improved the impairments induced by hypoperfusion possibly because of maintaining the integrity of BBB.

Histidine does not improve the blood flow velocity. In estimate the degree of hypoperfusion, the velocity of blood flow was measured. The velocity of blood flow in Histidine group had a tendency of increase, compared with that in Hypoperfusion group, but the difference was not significant (**Figure 2B**). This indicates that histidine could not increase the CBF directly to response to the hypoperfusion injury.

Although chronic cerebral hypoperfusion model was established successfully, this model could not exactly equate with the real situation of patients with atherosclerosis. The CCAs in human become narrow gradually because of the progression of atherosclerosis rather than abrupt stenosis in animal model. Additionally, the protective effects of histidine might be more efficacious if in a more severe hypoperfusion. A further study with longer observation period and more severe degree of hypoperfusion is needed to confirm the function of histidine.

## Conclusion

We observed that histidine could improve the hypoperfusion impairments in mice. This efficacy may involve the neurogenesis, astrocytes, and the integrity of the blood–brain barrier.

## Author Contributions

JS and HG designed the experiments and analyzed the data. JS, HG, and YW wrote the manuscript. JS, LY, DN, and QH performed the experiments.

## Conflict of Interest Statement

The authors declare that the research was conducted in the absence of any commercial or financial relationships that could be construed as a potential conflict of interest.

## Supplementary Material

FIGURE S1 The original figure of ZO-1 expression level in **Figure 3H**.

FIGURE S2 The original figure of β-actin expression level in **Figure 3H**.
