# Supplementary figures and images for: Histidine Alleviates Impairments Induced by Chronic Cerebral Hypoperfusion in Mice
Source: Front Physiol. 2018 Jun 7;9:662. doi: 10.3389/fphys.2018.00662 (PMC5999792; doi:10.3389/fphys.2018.00662)

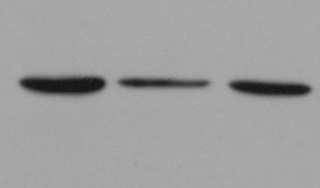

Supplement: FIGURE S1 — The original figure of ZO-1 expression level in Figure 3H. [file Image_1.JPEG]

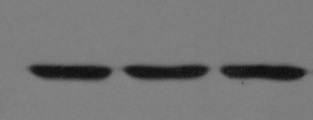

Supplement: FIGURE S2 — The original figure of β-actin expression level in Figure 3H. [file Image_2.JPEG]
